# Identifying etiologies of heart failure using non-contrast cardiac magnetic resonance imaging: cine imaging, T1 and T2 mapping, and texture analysis for T1 mapping

**DOI:** 10.3389/fcvm.2024.1471320

**Published:** 2025-01-21

**Authors:** Yasuo Amano, Yasuyuki Suzuki, Xiaoyan Tang, Chisato Ando

**Affiliations:** ^1^ Department of Radiology, Nihon University Hospital, Chiyoda-ku, Japan; ^2^ Department of Cardiology, Nihon University Hospital, Chiyoda-ku, Japan; ^3^ Department of Pathology, Nihon University Hospital, Chiyoda-ku, Japan; ^4^ Division of Radiological Technology, Nihon University Hospital, Chiyoda-ku, Japan

**Keywords:** heart failure, dilated cardiomyopathy, cardiac magnetic resonance, cine imaging, T1 mapping, texture analysis

## Abstract

**Objective:**

The aim of this retrospective study was to evaluate the usefulness of non-contrast cardiac magnetic resonance imaging, including cine imaging, T1 and T2 mapping, and texture analysis for T1 mapping, for identifying etiologies of heart failure (HF).

**Methods:**

Forty-seven patients with HF were examined using a 1.5 T scanner. Cine imaging parameters and native T1 and T2 values at the mid-septal segment were measured. Vertical run length nonuniformity, vertical gray level nonuniformity (vGLNU), wavelet energy LL(3) and HH (4) on T1 mapping were estimated at the mid-septal segment using open-access software. Late gadolinium enhancement was investigated to help diagnose the etiologies of HF. We used Kruscal-Wallis’ with a post-hoc Steel-Dwass' test, Wilcoxon signed-ranked test, Pearson's chai square test and receiver operator curve analysis (ROC) to assess the usefulness of non-contrast CMR for identifying etiologies of HF.

**Results:**

There were significant differences in left ventricular end-diastolic volume (LVEDV) indexed to body surface area (LVEDVi), left ventricular myocardial mass/LVEDV, native T1, and vGLNU between dilated cardiomyopathy (DCM), hypertensive cardiomyopathy (HC) and tachycardia-induced cardiomyopathies (TIC). DCM had higher T1 and lower vGLNU than HC. When compared with TIC, DCM showed significantly higher LVEDV and LVEDVi. ROC analysis revealed that LVEDV and vGLNU provided high specificity for differentiating DCM from the other etiologies.

**Conclusion:**

Native T1 mapping and its texture analysis may be valuable for differentiating between DCM and HC. Cine imaging can be useful for differentiating between DCM and TIC.

## 1 Introduction

Heart failure (HF) is a critical condition induced by a wide variety of etiologies, and HF can be a “pandemic” worldwide due to the rapid aging of the population (1). Because HF leads to rehospitalization or mortality, the medical cost is significantly increased by HF (1, 2). Identification of etiologies of HF may lead to appropriate treatments, and contribute to decreasing the mortality and medical cost of HF. Particularly, it is requested to differentiate dilated cardiomyopathy (DCM), which is often resistant to some treatments, from coronary artery disease and other types of cardiomyopathies, including hypertensive cardiomyopathy (HC) and tachycardia-induced cardiomyopathy (TIC), which are amenable the treatments that change the clinical course of the diseases (3–7).

Cardiac magnetic resonance (CMR) imaging provides reproducible measurement of left ventricular (LV) function and myocardial mass (LVM) and visualization of cardiac morphology with high spatial resolution and a wide range of views. Late gadolinium enhancement (LGE) CMR is particularly useful for differentiating various myocardial diseases showing HF and LV systolic dysfunction owing to its high potential to visualize replacement fibrosis (4, 5). LGE CMR is also valuable for risk stratification of DCM (8, 9). The drawback of LGE is the use of gadolinium-based contrast agents that are contraindicated to serious renal impairment associated with HF (10). Therefore, non-contrast CMR techniques for tissue characterization of the myocardium, including native T1 and T2 mapping, have been used to overcome the drawbacks of LGE (11–14). Texture analysis for CMR is used to identify some types of cardiomyopathies owing to its ability to quantify the tissue structure and inhomogeneity within digital images (15–19). To our knowledge, however, no previous studies have explored the identification of etiologies leading HF using the texture analysis for non-contrast CMR images.

Previous studies have demonstrated that DCM elevates T1 and T2 values of the myocardium, which are related to the prognosis of DCM (11–14). Thus, we hypothesized that T1 or T2 mapping may be valuable for differentiating DCM from other cardiomyopathies that are more treatable. Additionally, we assumed that either cine CMR parameters or texture analysis for T1 mapping could be utilized for this purpose. In this retrospective study, we sought to evaluate the usefulness of cine imaging, T1 and T2 mapping, and texture analysis for T1 mapping, for identifying etiologies of HF. Because texture analysis provides too many texture features that are difficult to handle in clinical practice and the use of in-house software may be difficult to generalize it, we used open-access software and focused on a few texture features.

## 2 Materials and methods

### 2.1 Study population

This retrospective study included consecutive patients with HF who underwent CMR between January 2018 and March 2024. An Institutional Review Board approved this study, and written informed consent was waived because of the retrospective fashion.

This study enrolled patients with HF irrespective of the HF types: reduced, mid-ranged, and preserved LV ejection fraction (LVEF). All patients underwent LGE CMR to help diagnose the etiologies of HF in this study. Exclusion criteria included: (1) patients with coronary artery diseases confirmed by coronary angiography or with ischemic LGE pattern (4, 5, 7), (2) patients with cardiac amyloidosis or sarcoidosis that had extracardiac manifestations and typical LGE patterns (7), (3) end-stage hypertrophic cardiomyopathy diagnosed clinically, pathologically, or by LGE CMR (20), (4) patients with serious valvular diseases diagnosed clinically and by echocardiography, (5) patients with congenital metabolic disorders, (6) patients with contraindication to gadolinium-based contrast agents due to allergy or serious renal impairment (10), and (7) patients with evident artifacts on CMR images due to respiratory or susceptibility artifact.

### 2.2 CMR acquisition

The CMR examinations were conducted with a 1.5 T imager (Ingenia, Philips Healthcare, Best, The Netherlands) with a 28-channel torso array coil. Cine balanced steady-state free precession (SSFP) was performed using the following imaging parameters: repetition time (TR), 3.2 ms; echo time (TE), 1.6 ms; flip angle (FA), 60°; in-plane resolution, 1.82 × 1.94 mm^2^; and slice thickness, 8 mm. A 5 s (3 s) 3 s modified Look-Locker inversion-recovery (MOLLI) was used for T1 mapping: a single-shot balanced SSFP readout was used with an inversion time of 159.5 ms after the first inversion recovery (IR) pulse, followed by 5 s data acquisition; a 3 s interval was set; and the 3 s data acquisition was performed after an inversion time of 350.0 ms after the second IR pulse. The imaging parameters of balanced SSFP used for MOLLI were as follows: TR, 2.8 ms; TE, 1.3 ms; FA, 35°; in-plane resolution, 2.00 × 1.97 mm^2^; and slice thickness, 10 mm. Gradient- and spin-echo imaging was used for T2 mapping with the scan parameters: TR, one cardiac cycle; TE, 20–60 ms every 10 ms (i.e., 5 echoes); FA, 90°; pixel size, 2.8 × 2.9 mm^2^; and slice thickness, 10 mm. Lastly, LGE CMR with phase sensitivity IR was performed with the typical parameters: TR, 6.1 ms; TE, 3 ms; FA, 25°; pixel size 1.59 × 1.79 mm^2^; and slice thickness, 10 mm. Cardiac gating, breath-holding, and parallel imaging (i.e., sensitivity encoding) techniques were applied to all imaging sequences. Cine and LGE imaging covered the whole LV cavity from the base to the apex on the short-axis view, while T1 and T2 mapping were acquired at the LV middle level on the short-axis view.

### 2.3 Image analysis

LVEF, LV end-diastolic volume (LVEDV), and LVM were measured using a commercial-based workstation (Ziostation2, Ziosoft, Tokyo, Japan) by a cardiologist with a 25 years' experience in cardiac imaging. Radiological technologists measured the myocardial T1 and T2 values at the mid-septum under supervision of the cardiologist.

One radiologist with a 27 years' experience in CMR assessed LGE visually and using the 6SD method. The radiologist conducted texture analysis for native T1 mapping using open-access software, MaZda (version 4.6; Institute of Electronics, Technical University of Lodz, Poland) (16, 17, 19, 21). An ROI was placed on the mid-septum by referring to the ROI employing native T1 measurement. The intensity range of the image under texture analysis was normalized by the default method (18): the intensity changes of the image ranged from 1 to 256. The 6 bits per pixel was used for image quantization. In this study, we evaluated 4 texture features, vertical run length nonuniformity (vRLNU), vertical gray level nonuniformity (vGLNU), wavelet energy (Wav En) LL(3) and Wav En HH(4) according to the previous studies (15–19).

### 2.4 Statistical analyses

LVEF, LVEDV, LVM, LVM/LVEDV, and LVEDV and LVM indexed to body surface area (LVEDVi and LVMi, respectively), native T1 and T2 values of the mid-septum, and 4 texture features on T1 mapping were compared between the etiologies of HF using the Kruskal-Wallis' test following the post-hoc Steel-Dwass' test. Additionally, the distribution of patients with elevated T1 or T2 values of the mid-septal myocardium was assessed between the etiologies of HF using Pearson's chai-square test. Because TIC has been considered as one of the causes of DCM (3, 22), we compared the parameters noted above between DCM and TIC using the Wilcoxon signed-ranked test. Receiver operator characteristic (ROC) analysis was used to determine the thresholds of the parameters to distinguish DCM from HC or TIC, because DCM is far more difficult to treat than HC and TIC. A *P*-value of less than 0.05 was considered indicative of statistical significance. EZR software was used for statistical analyses (22).

## 3 Results

### 3.1 Patients

**Figure 1:**
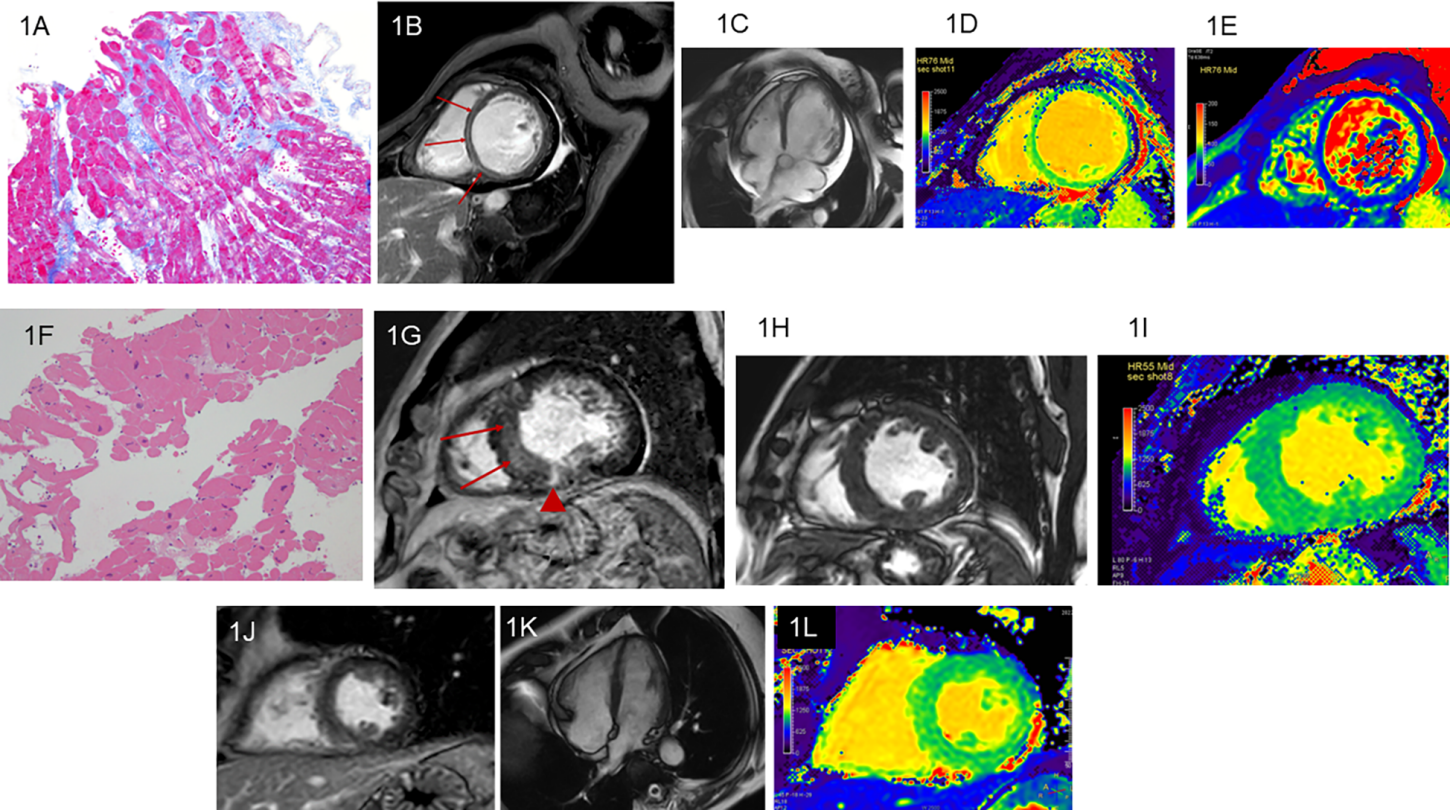
Dilated cardiomyopathy (DCM). A 63-year-old woman with heart failure (HF) and left ventricular (LV) ejection fraction (LVEF) of 11.3% measured by cine imaging. Endomyocardial biopsy reveals interstitial fibrosis, vacuole degeneration of myocyte, and myocardial disarray, which strongly indicate DCM **(A**: Masson stain**)**. The linear late gadolinium enhancement (LGE) is observed at the septal midwall (**B**; arrows). Cine imaging shows dilatation of the LV cavity and pericardial effusion **(C)**. The LV end-diastolic volume indexed to body surface area (LVEDVi) is 139.6 ml/mm^2^. Native T1 **(D)** and T2 **(E)** of the mid-septal myocardium increase to 1,172 ms and 53.0 ms, respectively. A 68-year-old man with hypertensive cardiomyopathy (HC) and reduced LVEF of 35.3%. Endomyocardial biopsy reveals myocyte swelling, enlargement of the myocyte nuclei, and periarterial fibrosis, whereas myocardial disarray is not found. Theses pathological findings are consistent with HC **(F**: hematoxylin eosin stain). The linear LGE is observed at the septum (**G**; arrows) and patch LGE is identified at the inferior myocardium **(G**; arrowhead). In this patient, LVEDVi is 99.5 ml/mm^2^ **(H)** and native T1 of the mid-septal myocardium is 1,048 ms **(I)**. A 68-year-old man with tachycardia-induced cardiomyopathy and LVEF of 44.0%. Endomyocardial biopsy is not performed. LGE is not observed **(J)**. The left ventricle seems normal in this patient **(K)**. LVEDVi is 44.1 ml/mm^2^ and native T1 of the mid-septal myocardium is 1,094 ms **(L)**.

A total of 47 patients with HF were included after the selection process outlined in Section 2.1. They included 37 men and 10 women with ages ranging from 34 to 95 years [mean, 64.0 years, standard deviation (SD), 13.4 years]. The final diagnoses determined as etiologies of HF were DCM in 25 patients (19 men; mean age, 65.2 years, SD 12.9 years), HC in 12 patients (10 men; mean age, 58.8 years, SD 12.2 years), TIC in 8 patients (6 men; mean age, 68.1 years, SD 14.1 years), aortic regurgitation identified by CMR in 1 patient, and mitral regurgitation identified by CMR in another patient. Patients with HC responded to treatments including anti-hypertensive agents, and those with TIC responded to treatments including atrial rate control. The pathological findings of endomyocardial biopsy were consistent with or not contradictory to the diagnoses of DCM in 11 patients and HC in 4 patients (Figures 1A,F). LGE were found in 31 of the 47 patients with HF (66.0%); 19 of the 25 patients with DCM (76.0%), 8 of the 12 patients with HC (66.7%), and 2 of the 8patients with TIC (25.0%) (Figures 1B,G,J). LGE patterns were nonischemic, linear or patch at the mid-wall or epicardial layer at the septal or the inferior myocardium in all etiologies of HF (Figures 1B,G).

### 3.2 Cine imaging parameters

**Figure 2:**
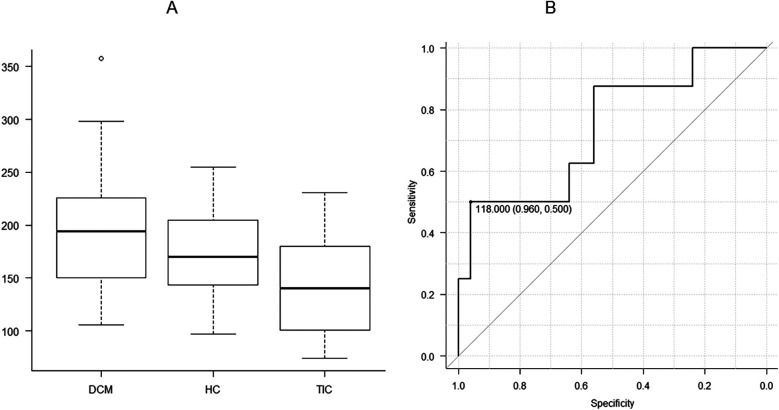
There are significant differences in left ventricular end-diastolic volume indexed to body surface area (LVEDVi) between three etiologies of heart failure, including dilated cardiomyopathy (DCM), hypertensive cardiomyopathy, and tachycardia-induced cardiomyopathy (TIC) **(A**; *P* < 0.05). When DCM and TIC are compared, receiver operating characteristic analysis shows that the threshold of 118.0 ml is appropriate for distinguishing between the 2 entities with the area under the curve of 0.740, a high specificity of 0.960 and a sensitivity of 0.500 **(B)**.

Table 1 summarizes the cine imaging parameters of DCM, HC, and TIC. There were significant differences in LVEDVi and LVM/LVEDV between the 3 etiologies of HF (*P* < 0.05 for both, Figures 1C,H,K, 2A). Although a non-significant tendency toward higher LVEDVi in DCM compared with TIC was found (*P* = 0.054), the post-hoc test did not reveal significant differences in any cine imaging parameters. No significant differences were observed for the other cine imaging parameters.

**Table 1:** Parameters of cine imaging, T1 and T2 mapping, and texture analysis of T1 mapping in the 3 etiologies of heart failure.

	DCM	HC	TIC	*P*
LVEF (%)	22.9 ± 9.9 (8.2–52.0)	32.2 ± 13.7 (13.0–59.0)	30.5 ± 13.4 (14.0–50.7)	0.126
T1 (ms)	1095.2 ± 51.8 (963.0–1200.0)	1054.7 ± 41.7 (987.0–1142.0)	1063.5 ± 48.6 (999.0–1146.0)	0.043^a^
T2 (ms)	51.8 ± 3.7 (41.5–58.5)	50.5 ± 4.7 (41.5–58.0)	51.0 ± 3.1 (46.0–54.5)	0.71
LVEDV (ml)	195.6 ± 57.4 (106.0–358.0)	172.9 ± 43.5 (97.0–255.5)	143.5 ± 50.1 (74.0–231.0)	0.11
LVEDVi (ml/m^2^)	114.3 ± 50.1 (63.1–203.4)	95.4 ± 24.6 (48.0–131.4)	83.0 ± 23.8 (48.6–121.6)	0.048^b^
LVM (g)	119.0 ± 36.3 (39.0–193.0)	142.7 ± 44.4 (84.0–240.0)	110.5 ± 32.0 (72.0–178.0)	0.16
LVMi (g/m^2^)	69.0 ± 18.5 (27.9–96.7)	77.4 ± 20.2 (48.1–123.7)	64.9 ± 17.8 (44.1–64.9)	0.42
LVM/LVEDV	0.64 ± 0.21 (0.23–0.97)	0.88 ± 0.32 (0.40–1.62)	0.82 ± 0.20 (0.53–1.09)	0.026^b^
vRLNU	82.4 ± 36.5 (21.9–137.0)	103.6 ± 52.5 (28.7–178.5)	78.5 ± 42.6 (20.7–136.9)	0.40
vGLNU	84.6 ± 30.0 (34.7–146.4)	128.5 ± 47.3 (43.8–186.6)	79.8 ± 33.0 (33.3–124.0)	0.027^a^
Wav En LL(3)	15,894 ± 3449 (11038–22482)	14,985 ± 3326 (11328–20768)	15,152 ± 3251 (11122–20516)	0.76
Wav En HH(4)	6.17 ± 3.49 (1.41–18.91)	7.49 ± 5.75 (1.95–25.1)	5.78 ± 2.53 (2.94–9.44)	0.80

DCM, dilated cardiomyopathy; HC, hypertensive cardiomyopathy; TIC, tachycardia-induced cardiomyopathy; LVEF, left ventricular ejection fraction; LVEDV, left ventricular end-diastolic volume; LVM, left ventricular myocardial mass; i means, indexed to body surface area; vRLNU, vertical run length nonuniformity; vGLNU, vertical gray level nonuniformity; Wav En, wavelet energy.

Parentheses represent the range of values.

^a^ There are significant differences between the 3 etiologies of heart failure (*P* < 0.05), and post-hoc test reveals the significant differences between DCM and HC (*P* < 0.05).

^b^ There are significant differences between the 3 etiologies of heart failure (*P* < 0.05).

When DCM and TIC were compared, LVEDV and LVEDVi were significantly higher in DCM than in TIC (*P* < 0.05 for both). ROC analysis revealed that LVEDV appropriate for differentiating between DCM and TIC was 118.0 ml [area under the curve [AUC], 0.740; 95% confidence interval [CI], 0.525–0.955; specificity, 0.960; sensitivity, 0.500; Figure 2B]. The LVEDVi appropriate for differentiating between DCM and TIC was 104.1 ml/m^2^ (AUC, 0.775; 95% CI, 0.585–0.965; specificity, 0.560; sensitivity, 0.875).

### 3.3 T1 and T2 mapping parameters

**Figure 3:**
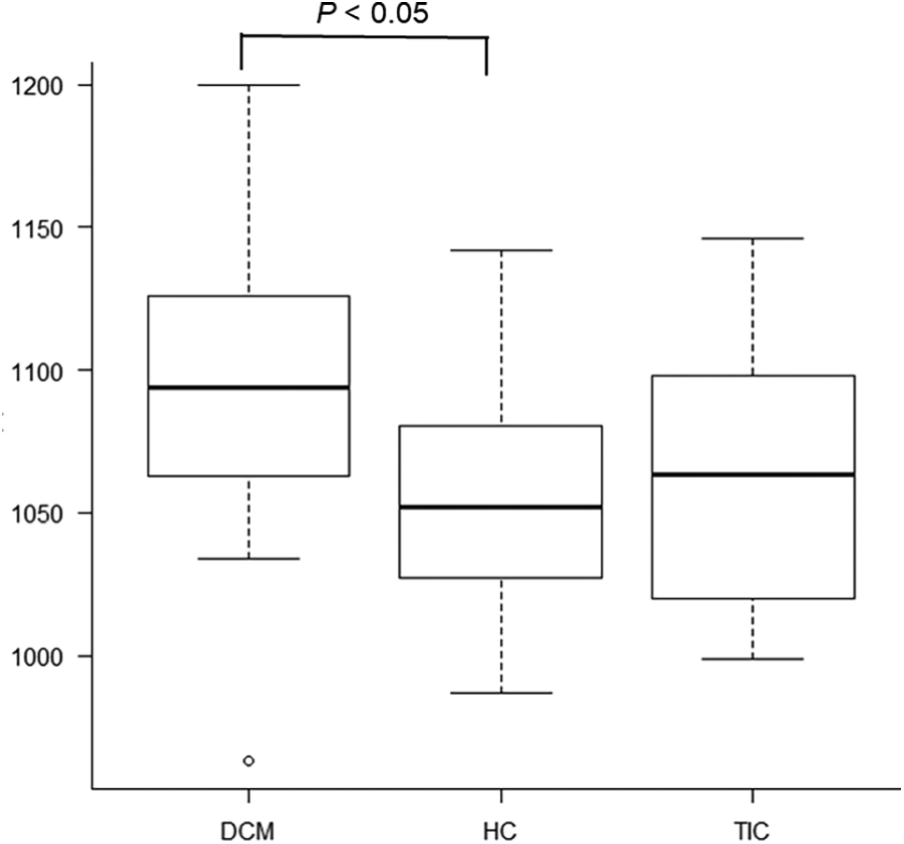
There are significant differences in native T1 values of the mid-septum between dilated cardiomyopathy (DCM), hypertensive cardiomyopathy (HC), and tachycardia-induced cardiomyopathy (*P* < 0.05). The pot-hoc test shows that DCM has significantly higher T1 than HC (*P* < 0.05).

Significant differences were observed in native T1 values of the mid-septum between the 3 etiologies of HF (*P* < 0.05, Figures 1D,I,L, 3, Table 1) but not in T2 values (*P* = 0.71, Table 1). The pot-hoc test showed that DCM had significantly higher T1 than HC (*P* < 0.05, Figure 3). The ROC analysis revealed that the native T1 appropriate for differentiating between DCM and HC was 1,082 ms (AUC, 0.742; 95% CI, 0.567–0.917; specificity, 0.600; sensitivity, 0.833).

When elevated T1 and T2 values were defined as those above the mean + 2SD, the elevated T1 was defined as 1111.0 ms or above (normal T1: mean, 1049.0 ms; SD, 31.0 ms) and the elevated T2 was 51.0 ms or above (normal T2: mean, 47.0 ms; SD, 2.0 ms), based on our healthy volunteer study. Elevated native T1 was observed in 8 (32.0%) of the 25 patients with DCM (Figure 1D), 1 (8.3%) of the 12 patients with HC, and 1 (12.5%) of the eight patients with TIC; there were no significant differences in the distribution of patients with elevated T1 between the 3 etiologies of HF (*P* = 0.20). Elevated T2 was observed in 16 (64.0%) of the 25 patients with DCM (Figure 1E), 5 (41.7%) of the 12 patients with HC, and 1 (12.5%) of the 8 patients with TIC. There were significant differences in the distribution of patients with elevated T2 between the 3 etiologies (*P* < 0.05).

### 3.4 Texture features for T1 mapping

**Figure 4:**
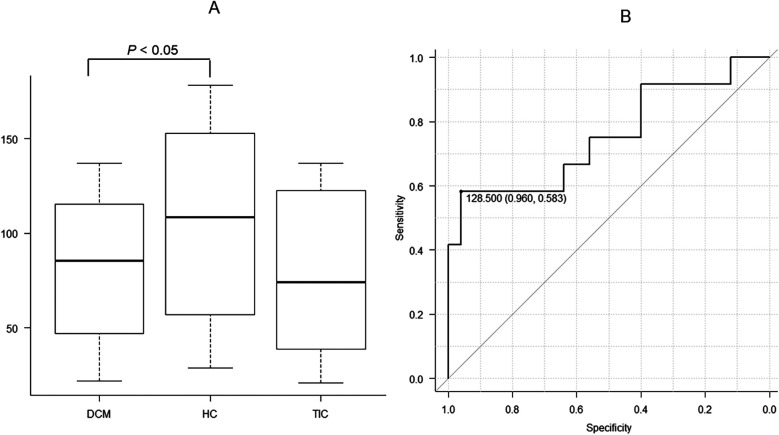
There are significant differences in vertical gray level nonuniformity (vGLNU) of T1 mapping at the mid-septum between dilated cardiomyopathy (DCM), hypertensive cardiomyopathy (HC), and tachycardia-induced cardiomyopathy **(A**; *P* < 0.05). The pot-hoc test shows that DCM has significantly lower vGLNU than HC (*P* < 0.05). Receiver operating characteristic analysis shows that the threshold of 128.5 is appropriate for distinguishing between DCM and HC with the area under the curve of 0.750, a high specificity of 0.960 and a high sensitivity of 0.583 **(B)**.

There were no significant differences in vRLNU, Wav En LL(3) and Wav EnHH(4) between the 3 etiologies of HF, whereas significant differences in vGLNU were investigated (*P* < 0.05, Figure 4A, Table 1). Additionally, DCM showed significantly lower vGLNU than HC (*P* < 0.05, Figure 4A). The ROC analysis revealed that vGLNU appropriate for differentiating between DCM and HC was 128.5 (AUC, 0.753; 95% CI, 0.563–0.944; specificity, 0.960; sensitivity, 0.583; Figure 4B).

## 4 Discussion

The present study demonstrated significant differences in LVEDVi, LVM/LVEDV, native T1, and vGLNU between 3 etiologies of HF: DCM, HC, and TIC. DCM showed significantly higher T1 and lower vGLNU than HC, and DCM tended to exhibit higher LVEDV and LVEDVi compared with TIC. Therefore, combined use of non-contrast CMR techniques, including cine imaging, T1 mapping, and texture analysis for T1 mapping, may identify the underlying etiologies of HF.

The increase in LVEDVi, a simple cine imaging parameter, suggests DCM as the etiology of HF. The percentage of the DCM patients with LGE (76.0%) in our study was larger than in previous studies (8, 9) and almost identical to those of other studies (6, 23), indicating that DCM showing HF may have replacement fibrosis frequently. The elevated T1 and T2 might reflect diffuse fibrosis, increased water contents, and chronic inflammation in DCM, probably leading to the dilatation of the LV cavity (12, 14, 24, 25). The present study indicates that the LVEDV of 118.0 ml distinguished DCM from TIC with a high specificity of 0.960, which indicate the LVEDV contributes to the diagnosis of DCM. Orlov et al. (23) reported that DCM has dilated LV cavities compared with TIC.

Native T1 was significantly higher in DCM than in HC. This result indicated that myocardial fibrosis may be diffuse in DCM than in HC. Although there were significant differences in the distribution of patients with elevated T2 between the 3 etiologies of HF, T2 values did not differ significantly. This may be attributed to the definition of elevated T2 in this study (i.e., >mean + 2SD). In addition, myocardial T2 might be prolonged in patients with HF, regardless of its etiologies (24, 26).

Unexpectedly, vGLNU was lower in DCM than in HC. The vGLNU of 128.5 distinguished DCM from HC with a high specificity of 0.960. Therefore, vGLNU may contribute to the identification of DCM as the etiology of HF. The vGLNU represents the inhomogeneity of the pixel gray level distribution in the images (15, 16, 18). These results indicate that DCM includes myocardial fibrosis, but less inhomogeneous tissue structures than HC. Although the pathohistological examinations revealed myocardial disarray in the patients with DCM but not in those with HC in our study (Figures 1A,F), a previous report indicates that HC contains various types of myocardial fibrosis and myocyte enlargement, which may induce tissue inhomogeneity (27). Further study will be required to correlate between vGLNU on T1 mapping and histological findings in various etiologies of HF.

TIC exhibited normal T1 and T2 values of the mid-septum with only 2 of the 8 patients showing LGE. The percentage of TIC patients with LGE (i.e., 25.0%) was a little higher than that of the previous studies (6, 23, 28), probably because all patients with TIC and HF might have long periods of illness and because of the study's limited population. The causes of TIC, such as atrial fibrillation, supraventricular tachycardia, and premature ventricular contraction, might contribute to the presence of LGE.

Advantages of this texture analysis were the retrospective use of open-access software, but not in-house software, and the focus on only 4 texture features. The combined use of texture analysis and artificial intelligence can select more texture features, allowing distinction of the etiologies of HF (15), but it may be difficult to handle so many features and correlate them with histopathological or clinical findings in the daily routine. In addition, it has not been established how to validate artificial intelligence with clinical data. We selected and analyzed the 4 features that were believed to correspond to tissue alternations and inhomogeneities (16–19). Consequently, there were significant differences in vGLNU between DCM, HC, and TIC, and DCM exhibited significantly lower vGLNU than did HC.

There were some limitations in this study. First, the patient population was small partly because of the strict inclusion criteria. This study was a single-center retrospective study. These factors may reduce statistical power, bias the study sample, and affect the generalization of the present results. Second, the endomyocardial biopsy did not always confirm the etiologies of HF because of sampling error associated with the right ventricular catheter examination. The spatial resolution of CMR was much coarser than that of the biopsied specimen. Third, we did not analyze all texture features available through MaZda software (21). The use of artificial intelligence may extract more fruitful texture features appropriate for distinguishing etiologies of HF (15, 29). However, it may be difficult to manage so many texture features in daily clinical scenarios. Again, it has not been established how to validate artificial intelligence with clinical data. Lastly, the relationship between the non-contrast CMR and prognosis was beyond the scope of this study. Because our patients with HF tended to have LGE in each etiology more frequently than those in previous studies in each etiology (8, 9, 20, 23, 28), we assumed that their prognosis was unfavorable. The relation between prognosis and non-CMR parameters or texture features should be determined in each etiology of HF.

In conclusion, non-contrast CMR, including cine imaging, T1 mapping and texture analysis for T1 mapping, provides quantitative parameters to identify the etiologies of HF. The measurements of native T1 and vGLNU are useful for distinguishing DCM from HC, particularly with a high specificity for vGLNU. This study also suggests that DCM has significant dilatation of the LV cavity compared with TIC.

## Data Availability

The original contributions presented in the study are included in the article/Supplementary Material, further inquiries can be directed to the corresponding author.
